# Supplementary material for: Identification and Analysis of Fungal-Specific Regions in the Aspergillus fumigatus Cu Exporter CrpA That Are Essential for Cu Resistance but Not for Virulence
Source: Int J Mol Sci. 2023 Feb 13;24(4):3705. doi: 10.3390/ijms24043705 (PMC9966986; doi:10.3390/ijms24043705)
Supplement: Supplementary file 1 [file ijms-24-03705-s001.zip › ijms-2062205-supplementary.pdf]

Supplementary Materials.

Table S1: Strains used in this study

Strain	Genotype	Source
CEA17 KU80 (wt)	*pyrG1*, *ΔakuB::pyrG, pyrG1*	(1)
Δ*CrpA*	*pyrG1, ΔakuB::pyrG, pyrG1, ΔAFUB_036430::pyrG*	(2)
CrpA_dN	*ΔAFUB_036430::pyrG1*<br>*AFUB_036430_dN_GFP::Phleo*	This study
CrpA_dmid	*ΔAFUB_036430::pyrG1*<br>*AFUB_036430_dmid_GFP::Phleo*	This study
CrpA_cont	*ΔAFUB_036430::pyrG1*<br>*AFUB_036430_GFP::Phleo*	This study
*CrpA_dN**	*ΔAFUB_036430::pyrG1*<br>*AFUB_036430_dN_GFP::Phleo*<br>*AMA1-pyr4*	This study
*CrpA_dmid**	*ΔAFUB_036430::pyrG1*<br>*AFUB_036430_dmid_GFP::Phleo*<br>*AMA1-pyr4*	This study
*CrpA_cont**	*ΔAFUB_036430::pyrG1*<br>*AFUB_036430_GFP::Phleo*<br>*AMA1-pyr4*	This study

Table S2: Primers used in this study

Primer	Sequence 5'-3'
dNcrpA_5Flank_fwd	CCATATGGTCGACCTGCAGGCGGCCGGCGCGCCGAGCATAACGGACAA GGG
dNcrpA_5Flank_rev	ATGCACAAGTCATGGTTATCAGTGGCAACTTTC
dNcrpA_dNcrpA_fwd	GCCACTGATAACCATGACTTGTGCATCGTGC
dNcrpA_dNcrpA_rev	CCTTGCTCACCATAGACATAGACCGACCCAAC
dNcrpA_GFPterm_fwd	TCGGTCTATGTCTATGGTGAGCAAGGGCGAG
dNcrpA_GFPterm_rev	TACAAGGGAATTCAAGAAGGATTACCTCTAAACAAGTGTACC
dNcrpA_phleo_fwd	GGTAATCCTTCTTGAATTCCCTTGTATCTCTACAC
dNcrpA_phleo_rev	TCAACCAACTCCTAAGAAGGATTACCTCTAAACAAG
dNcrpA_3Flank_fwd	GGTAATCCTTCTTAGGAGTTGGTTGAGAGTTTC
dNcrpA_3Flank_rev	CACTGGGCGAATTGGGCCCGACGTCGGCGCGCCAAAAGGAGCTTGTTT ACGAG
dNcrpA_vector_fwd	GGCGCGCCGACGTCGGGCCCAATTCG

dNcrpA_vector_rev	GGCGCGCCGGCCGCCTGCAGGTCGAC
dMidcrpA_dpart1_fwd	TGGTCGACCTGCAGGCGGCCATGGCTACGGAAACGAGG
dMidcrpA_dpart1_rev	CGGATCCTGAGCCACTAAGTGAGTAGATCTCTTTCATGG
dMidcrpA_part2_fwd	CTACTCACTTAGTGGCTCAGGATCCGGGAGCGGTTCTGGCTCGGGAAGT GGGTCATTCGGCAGCATGAATCTTC
dMidcrpA_part2_rev	GGCGAATTGGGCCCGACGTCAGACATAGACCGACCCAAC
dMidcrpA_Vector_fwd	GACGTCGGGCCCAATTCG
dMidcrpA_Vector_rev	GGCCGCCTGCAGGTCGAC
dMidcrpA_5Flank_fwd	TGGTCGACCTGCAGGCGGCCGGCGCGCCGAGCATAACGGACAAGGG
dMidcrpA_5Flank_rev	CCGTAGCCATGGTTATCAGTGGCAACTTTC
dMidcrpA_dMidcrpA_fwd	ACTGATAACCATGGCTACGGAAACGAGG
dMidcrpA_dMidcrpA_rev	TGCTCACCATAGACATAGACCGACCCAAC
dMidcrpA_GFP_fwd	GTCTATGTCTATGGTGAGCAAGGGCGAG
dMidcrpA_GFP_rev	AAGGGAATTCAAGAAGGATTACCTCTAAACAAGTGTACC
dMidcrpA_Phleo_fwd	AATCCTTCTTGAATTCCCTTGTATCTCTACAC
dMidcrpA_Phleo_rev	ACCAACTCCTAAGAAGGATTACCTCTAAACAAG
dMidcrpA_3Flank_fwd	AATCCTTCTTAGGAGTTGGTTGAGAGTTTC
dMidcrpA_3Flank_rev	GGCGAATTGGGCCCGACGTCGGCGCGCCAAAAGGAGCTTGTTTACGAG
CrpA_control_5F_fwd	CCATATGGTCGACCTGCAGGCGGCCGGCGCGCCGAGCATAACGGACAA GGG
CrpA_control_5F_rev	TTCCGTAGCCATGGTTATCAGTGGCAACTTTC
CrpA_control_crpA_fwd	GCCACTGATAACCATGGCTACGGAAACGAGG
CrpA_control_crpA_rev	CCTTGCTCACCATAGACATAGACCGACCCAAC
CrpA_control_GFP_fwd	CGGTCTATGTCTATGGTGAGCAAGGGCGAG
CrpA_control_GFP_rev	TACAAGGGAATTCAAGAAGGATTACCTCTAAACAAGTG
CrpA_control_phleo_fwd	GTAATCCTTCTTGAATTCCCTTGTATCTCTACAC
CrpA_control_phleo_rev	TCAACCAACTCCTAAGAAGGATTACCTCTAAACAAG
CrpA_control_3Flank_fwd	GTAATCCTTCTTAGGAGTTGGTTGAGAGTTTC
CrpA_control_3Flank_rev	CACTGGGCGAATTGGGCCCGACGTCGGCGCGCCAAAAGGAGCTTGTTT ACGAG
CrpA_control_vec_fwd	GACGTCGGGCCCAATTCGC
CrpA_control_vec_rev	GGCCGCCTGCAGGTCGAC

Transformation of *Aspergillus fumigatus*. The recipient *A. fumigatus* strain CEA17 KU80 was spread on YAG solid medium and incubated for 48 h at 37°C. Spores were collected, suspended in DDW and counted. $5x10^9$ spores were suspended in 50 ml YAG/YAG UU liquid medium and incubated with shaking for 6 h at 37°C. Spores were then centrifuged for 3 minutes at 1400 g, suspended in 20 ml lytic mix and incubated for two hours at 30°C to digest the cell wall and generate protoplasts. The protoplasts were centrifuged for 3 minutes at 1400 g and washed in 20 ml of solution C. After another 3 minutes of centrifugation at 1400 g, protoplasts were suspended in 500 µl of solution E with 5 µl 0.1 M DDT and incubated overnight at 4°C.

The solutions used are listed below:

Solution A: 0.1 M citric acid and 1.1 M KCl were titrated with KOH to pH 5.8.

Solution B: 0.2% (v/v) vitamin mix, 0.72% (w/v) glucose, 2% (w/v) sucrose, 1% (w/v) yeast extract.

Solution C: 0.6 M KCl, 0.05 M citric acid pH 5.8, 1% (w/v) glucose.

Solution D: 25% PEG 8000, 10 mM Tris-HCl pH 7.5, 100 mM $CaCl_2$, 0.6 M KCl.

Solution E: 100 mM $CaCl_2$, 0.6 M KCl, 10 mM Tris-HCl pH 7.5, 0.1 M DTT.

Lytic (protoplasting) mix: 50% solution A, 50% solution B, 10 mM $MgSO_4$, 1% BSA, 1% driselase (Sigma-Aldrich), 0.5% lysing enzymes (Sigma-Aldrich).

For the transformation reaction, 25 µl of the protoplast suspension was suspended with 1 µg of the prepared deletion construct and with 12.5 µl of solution D and incubated for 20 minutes on ice. Afterwards, another 180 µl of solution D were added and the suspension was incubated at room temperature for another 20 minutes. Next, 220 µl from the protoplast suspension were suspended into 4 ml MMVUU-Sorbitol-TOP medium that was heated to 42°C, and spread on top of 15 ml MMVUU-Sorbitol solid medium plates. Plates were incubated at room temperature for 10 h. Then, 10 ml of MMVUU-Sorbitol-Top were mixed with 20 µg/ml phleomycin and poured on top of the transformation plates. Plates were incubated for another 12 h at room temperature and then at least 48 h more at 37°C.

Gibson assembly. Assembly of DNA fragments was made using NEBuilder HiFi DNA Assembly Master Mix (New England Biolabs, Inc.). 0.04-0.2 pmols of each flank in a maximum final volume of 10 µl was added to 10 µl of the NEBuilder HiFi DNA Assembly Master Mix and ultra-pure DDW completed to 20 µl total reaction volume. The mix was incubated in a PCR machine at 50°C for 1 h. The plasmid was diluted 1:3 and transformed by electroporation into *E. coli DH10B* strain. Transformed cells were spread on LB solid medium (supplemented with 100 µg/ml Ampicillin as

a selection marker and Xgal + IPTG) and incubated overnight. After 24 h, white colonies were picked to LB liquid medium (supplemented with 100 µg/ml Ampicillin) and incubated overnight at 37°C. Purification of the plasmid was made using the Wizard® Plus SV Minipreps DNA Purification System (Promega Inc.) commercial kit and validation was made by sequencing and restriction enzyme analysis. Linearization prior to transformation was made using *AscI*.

Generation of *A. fumigatus* strains used in this study.

CrpA_dN construct was made by joining six PCR fragments the Gibson assembly method: 5'flank (using primers dNcrpA_5Flank_fwd and dNcrpA_5Flank_rev), ΔNcrpA (using primers dNcrpA_dNcrpA_fwd and dNcrpA_dNcrpA_rev), GFP (using primers dNcrpA_GFPterm_fwd and dNcrpA_GFPterm_rev), Phleo cassette (using primers dNcrpA_phleo_fwd and dNcrpA_phleo_rev), 3'flank (using primers dNcrpA_3Flank_fwd and dNcrpA_3Flank_rev) and pUC19 vector (using primers dNcrpA_vector_fwd and dNcrpA_vector_rev). A unique *AscI* restriction site, introduced into primers (dNcrpA_5Flank_fwd and dNcrpA_3Flank_rev), was later used to linearize the construct for transformation. Transformation of the construct into *A. fumigatus* was performed as described above. Verification of correct integration of the construct into the desired locus was performed by PCR as shown in Supplemental Figures 1 and 2. Six correctly integrated colonies were identified and shown to express GFP-ΔNCrpA on MMVUU containing 2.5 µM Cu and show the same level of Cu sensitivity. Colony 1 was used in further studies.

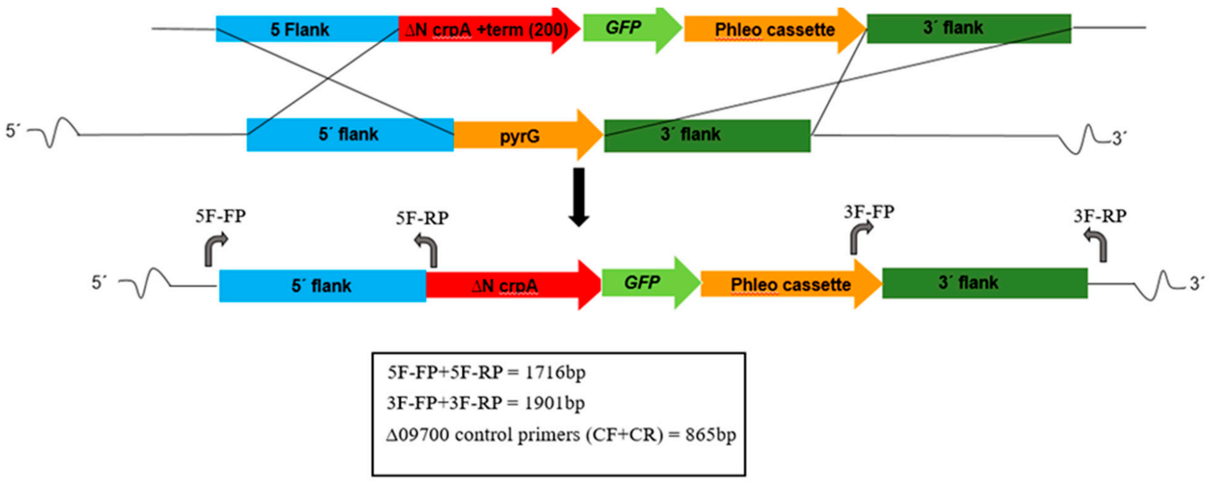

Supplemental Figure S1. CrpA_dN construct and visualization of integration into the genome.

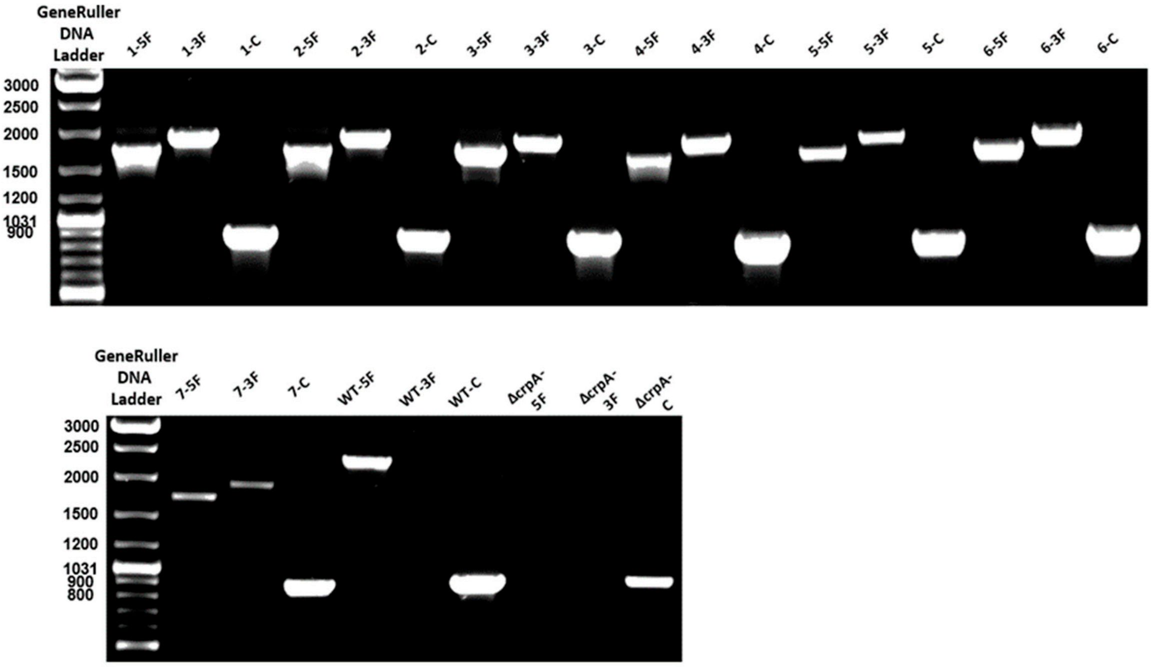

Supplemental Figure S2. *CrpA_dN* transformant validation by colony PCR was made with the primers listed in Supplemental Figure 5. Colonies 1, 2, 3, 4, 5 and 6 were taken for further analysis.

CrpA_dmid was constructed by using an intermediate plasmid, CrpA_dmid_part1, which was made by joining three PCR fragments using the Gibson assembly method: ΔcrpAp1 (using primers dMidcrpA_dpart1_fwd and dMidcrpA_dpart1_rev), ΔcrpAp2 (using primers

dMidcrpA_part2_fwd and dMidcrpA_part2_rev) and pUC19 vector (using primers dMidcrpA_Vector_fwd and dMidcrpA_Vector_rev). The final plasmid was made by joining six PCR fragments: 5'flank (using primers dMidcrpA_5Flank_fwd and dMidcrpA_5Flank_rev), ΔcrpAp1p2 (using primers dMidcrpA_dMidcrpA_fwd and dMidcrpA_dMidcrpA_rev) that was amplified from the intermediate plasmid CrpA_dmid_part1, GFP (using primers dMidcrpA_GFP_fwd and dMidcrpA_GFP_rev), Phleo cassette (using primers dMidcrpA_Phleo_fwd and dMidcrpA_Phleo_rev), 3'flank (using primers dMidcrpA_3Flank_fwd and dMidcrpA_3Flank_rev) and pUC19 vector (using primers dMidcrpA_Vector_fwd and dMidcrpA_Vector_rev). A unique *AscI* restriction site, introduced into primers (dMidcrpA_5Flank_fwd and dMidcrpA_3Flank_rev), was later used to linearize the construct for transformation. Transformation of the construct into *A. fumigatus* was performed as described above. Verification of correct integration of the construct into the desired locus was performed by PCR as shown in Supplemental Figures 3 and 4. Three correctly integrated colonies were identified and shown to express GFP-CrpA_dMid on MMVUU containing 2.5 µM Cu, and show the same level of Cu sensitivity. Colony 1 was used in further studies.

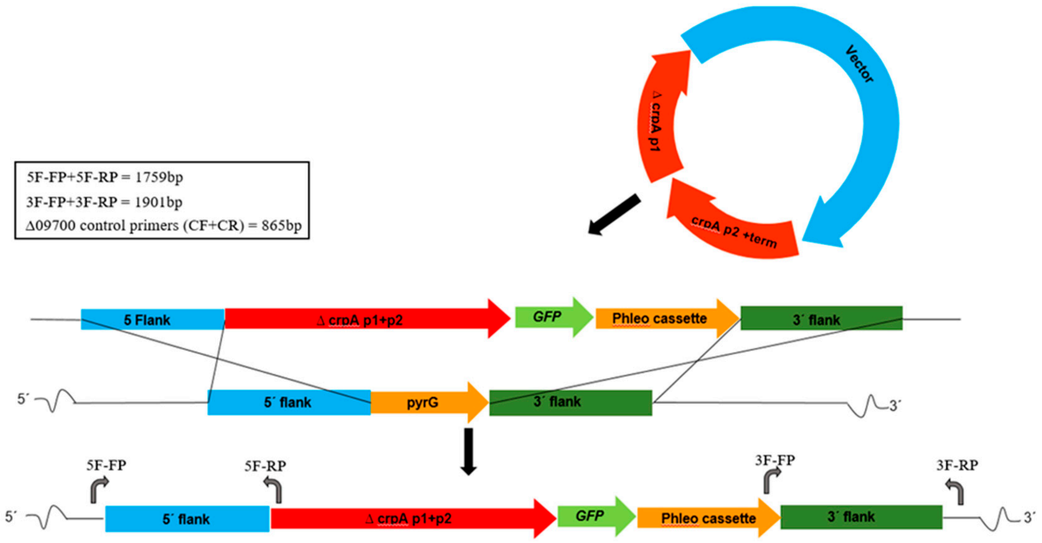

Supplemental Figure S3. CrpA_dMid construct and visualization of integration into the genome.

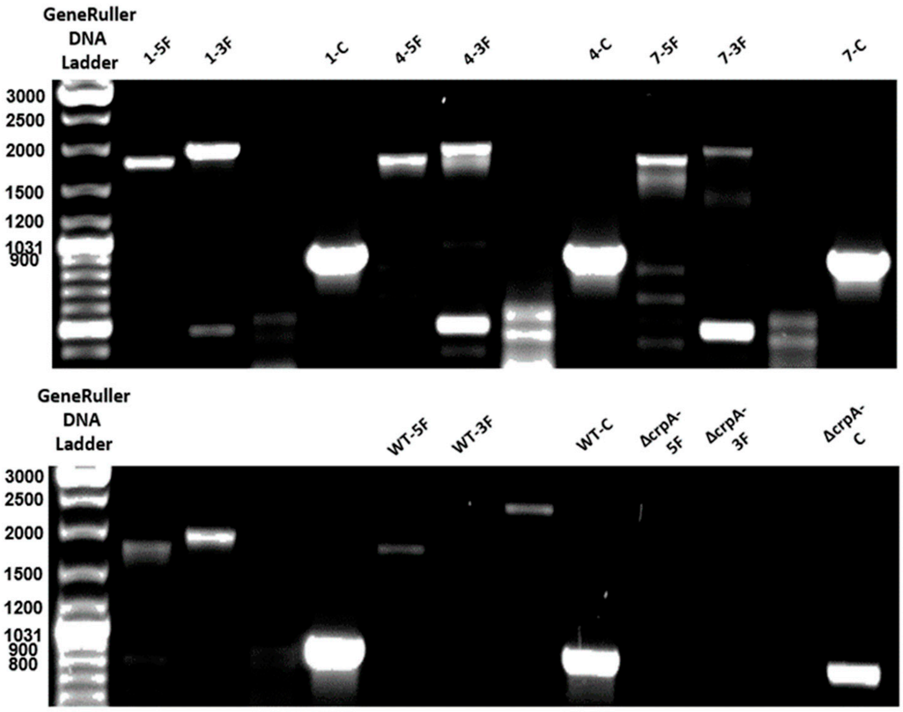

Supplemental Figure S4. Validation of *CrpA_dMid* transformants by colony PCR was made with the primers mentioned in Supplemental Figure 10B. Colonies 1, 4 and 7 were taken for further analysis.

CrpA_cont was constructed by joining six PCR fragments: 5'flank (using primers CrpA_control_5F_fwd and CrpA_control_5F_rev), crpA_control (using primers CrpA_control_crpA_fwd and CrpA_control_crpA_rev), GFP (using primers CrpA_control_GFP_fwd and CrpA_control_GFP_rev), Phleomycin cassette (using primers CrpA_control_phleo_fwd and CrpA_control_phleo_rev), 3'flank (using primers CrpA_control_3Flank_fwd and CrpA_control_3Flank_rev) and pUC19 vector (using primers CrpA_control_vec_fwd and CrpA_control_vec_rev). A unique *AscI* restriction site, introduced into primers (CrpA_control_5F_fwd and CrpA_control_3Flank_rev), was later used to linearize the construct for transformation. Primer sequences are detailed in supplementary table 2. All PCR reactions mentioned above were conducted using Phusion high-fidelity PCR system (ThermoFisher scientific). Transformation of the construct into *A. fumigatus* was performed as described above. Verification of correct integration of the construct into the desired locus was performed by PCR as shown in Supplemental Figures 5 and 6. One correctly integrated colonies were identified and shown to express GFP-CrpA on MMVUU containing 2.5 µM Cu. Colony 2 was used in further studies.

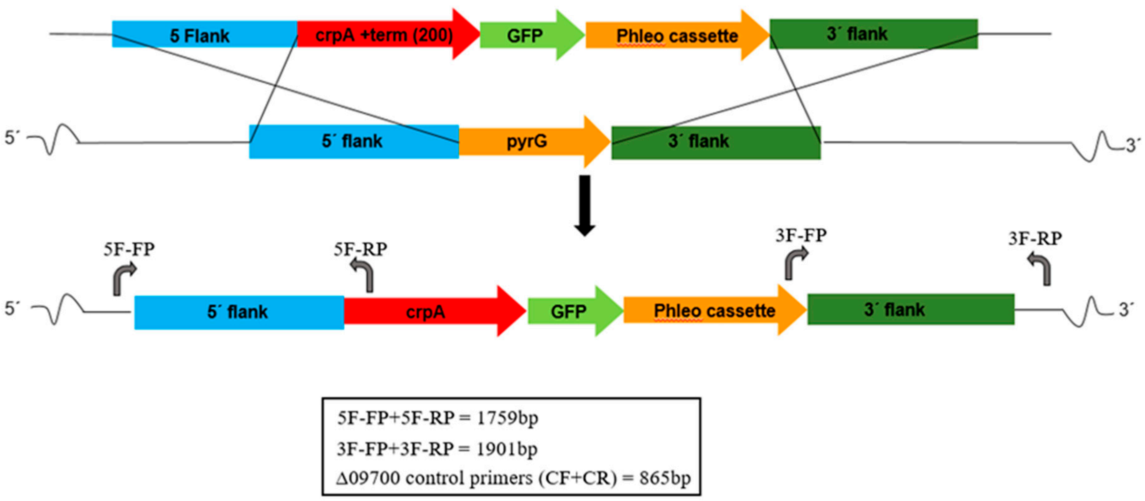

Supplemental Figure S5. CrpA_cont construct and visualization of integration into the genome.

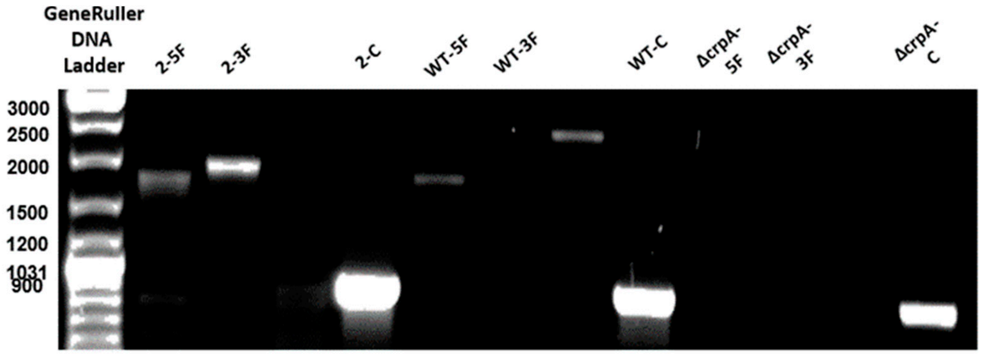

Supplemental Figure S6. *CrpA_cont* transformant validation by colony PCR was made with the primers listed in Supplemental Figure 10C. Colony 2 was taken for further analysis.

Generation of *CrpA_dN*, CrpA_dmid and *CrpA_cont** strains**. *CrpA_dN*, CrpA_dmid** and *CrpA_cont** uracil prototroph strains used in the virulence analysis were generated by transforming *CrpA_dN, CrpA_dmid* and *CrpA_cont* uracil auxotroph strains with the pAMA-1 self-replicating plasmid (3) containing *pyr4*, onto MMV-sorbitol plates. Following transformation, the strains were tested on MMV agar plates containing increasing concentrations of Cu. They showed the same Cu susceptibilities as their corresponding auxotrophic strains: CrpA_dmid* was very sensitive to high concentrations of Cu, and the CrpA_dN* strain was slightly more sensitive to high Cu than the CrpA_cont* strain, indicating that acquisition of uracil prototrophy did not affect Cu sensitivity (Supplemental Figure 7).

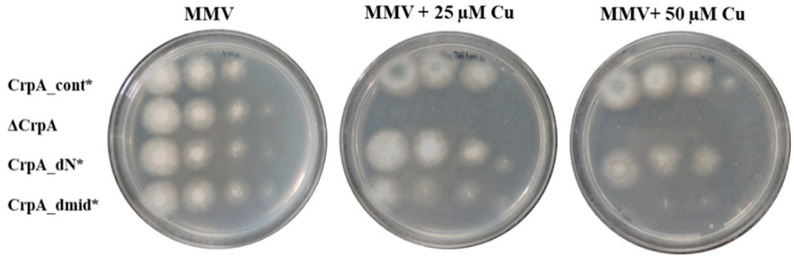

Supplemental Figure S7. *CrpA_dN*, CrpA_dmid** and *CrpA_cont** strains maintain the same sensitivity to Cu following uracil prototrophy. Strains were grown on MMV agar plates supplemented with increasing concentrations of Cu. Conidial droplets containing increasing numbers of conidia (10, 100, 1000, and 10,000 from right to left) were placed on the plates and incubated for 72 h at 37°C.

REFERENCES

1. da Silva Ferreira ME, Kress MR, Savoldi M, Goldman MH, Hartl A, Heinekamp T, Brakhage AA, Goldman GH. 2006. The akuB(KU80) mutant deficient for nonhomologous end joining is a powerful tool for analyzing pathogenicity in *Aspergillus fumigatus*. Eukaryot Cell 5:207-11.
2. Wiemann P, Perevitsky A, Lim FY, Shadkchan Y, Knox BP, Landero Figueora JA, Choera T, Niu M, Steinberger AJ, Wuthrich M, Idol RA, Klein BS, Dinauer MC, Huttenlocher A, Osherov N, Keller NP. 2017. *Aspergillus fumigatus* Copper Export Machinery and Reactive Oxygen Intermediate Defense Counter Host Copper-Mediated Oxidative Antimicrobial Offense. Cell Rep 19:2174-2176.
3. Osherov N, Kontoyiannis DP, Romans A, May GS. 2001. Resistance to itraconazole in *Aspergillus nidulans* and *Aspergillus fumigatus* is conferred by extra copies of the *A. nidulans* P-450 14alpha-demethylase gene, *pdmA*. J Antimicrob Chemother 48:75-81.
